# Enhancing Tabletop X-Ray Phase Contrast Imaging with Nano-Fabrication

**DOI:** 10.1038/srep13581

**Published:** 2015-08-28

**Authors:** Houxun Miao, Andrew A. Gomella, Katherine J. Harmon, Eric E. Bennett, Nicholas Chedid, Sami Znati, Alireza Panna, Barbara A. Foster, Priya Bhandarkar, Han Wen

**Affiliations:** 1 Imaging Physics Laboratory, Biochemistry and Biophysics Center, National Heart, Lung and Blood Institute, National Institutes of Health, Bethesda, MD 20892; 2 Breast Imaging Center, Walter Reed National Military Medical Center, Bethesda, MD 20889.

## Abstract

X-ray phase-contrast imaging is a promising approach for improving soft-tissue contrast and lowering radiation dose in biomedical applications. While current tabletop imaging systems adapt to common x-ray tubes and large-area detectors by employing absorptive elements such as absorption gratings or monolithic crystals to filter the beam, we developed nanometric phase gratings which enable tabletop x-ray far-field interferometry with only phase-shifting elements, leading to a substantial enhancement in the performance of phase contrast imaging. In a general sense the method transfers the demands on the spatial coherence of the x-ray source and the detector resolution to the feature size of x-ray phase masks. We demonstrate its capabilities in hard x-ray imaging experiments at a fraction of clinical dose levels and present comparisons with the existing Talbot-Lau interferometer and with conventional digital radiography.

While conventional x-ray images indicate the amount of radiation energy deposited in materials according to the density distribution, phase contrast techniques are sensitive to the variation of the refractive index in the sample similar to the phase contrast mode of a light microscope. Refractive index variations lead to the bending and scattering of the x-ray wavefront which are detected by these techniques without depositing substantial energy in the sample^1^. Some of the first x-ray phase contrast images were diffraction enhanced images using Bragg analyzer crystals^2^,^3^ and free space propagation of spatially coherent beams^4^,^5^. At the same time monolithic crystal interferometers^6^ were extended to obtain high-contrast images of soft tissue samples^1^. Subsequently, x-ray grating interferometers were proposed and then realized^7^,^8^,^9^,^10^ for phase contrast imaging. Diffraction of a coherent beam by random phase objects and sharp edges also lead to speckle-based techniques^11^,^12^,^13^.

Common x-ray tubes are the choice for table-top systems, but their low spatial and temporal coherence has required additional filtering by absorptive elements such as single crystals that select a narrow incident angle for a given photon energy^2^, absorption masks^14^ or absorption gratings^7^,^10^ that improve lateral coherence. Additionally, polychromatic techniques often sense wavefront distortion from micron-scale intensity fringes, which requires high enough resolution on the part of the detector to resolve them. For conventional area detectors with typical resolutions of 50 μm or above this is solved by using an analyzer filter^7^,^10^,^14^. Thus just a fraction of the photon flux reaches the detector.

We show that it is possible to overcome this limitation and at the same time substantially elevate the sensitivity of tabletop systems with phase-shifting elements whose features are comparable in size to the lateral coherence of the x-ray source. The method is a polychromatic far-field interferometer (PFI) consisting of three phase gratings (Fig. 1). A phase grating imprints a periodic phase pattern on the x-ray wavefront. Recent progress in nano-fabrication provided hard x-ray phase gratings with periods down to 200 nm^15^, which approaches the lateral coherence of x-ray tube sources. Such gratings enabled the method to be realized in a table-top system. We demonstrate its capabilities in imaging experiments of biological specimens and a standard breast-mimicking phantom. We compare its performance with the existing Talbot-Lau interferometer for compact sources and with a state-of-the-art digital mammography scanner. To introduce its physical mechanism we describe a theoretical model with closed-form expressions for the key parameters of the system, which we verify experimentally.

## Results

**Figure 1:**
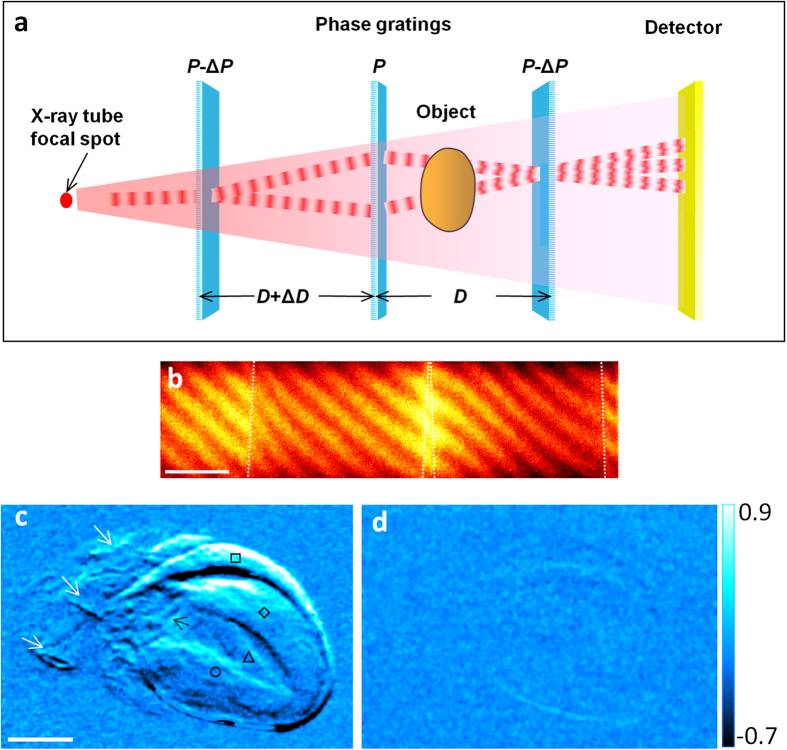
Schematic illustration of the hard x-ray polychromatic far-field interferometer. (**a**) The imaging system consists of an x-ray tube emitting a polychromatic cone beam, three phase gratings and an area detector. The grating period *P* of 200 nm is comparable to the lateral coherence length of the x-ray tube at the first grating. A virtual narrow fan beam within the cone beam forms a polychromatic interferometer through grating diffraction as illustrated by the red waves. (**b**) Interferogram from the entire cone beam. Fringe distortion indicates the refractive index of water-filled Teflon tubes placed in the beam. The borders of the two tube segments are indicated by the dashed lines. The fringe contrast is optimized by adjusting the effective periods of the first and third gratings through tilting (Δ*P*) and adjusting the difference in the inter-grating spacings (Δ*D*). Scale bar is 5 mm. (**c**) A single-projection differential phase image of an unstained mouse heart specimen immersed in water taken with the PFI at 1.08 mGray entrance surface dose. The visible structures are the right ventricular wall (square), the septum (diamond), a papillary muscle column (triangle), the left ventricular wall (circle), the mitral valve (black arrow), and blood vessels (white arrows). Scale bar is 2 mm. (**d**) Differential phase image from a Talbot-Lau interferometer at 2.16 mGray entrance surface dose. Differential phase image is the phase shift of the interference fringes and proportional to the slope of the x-ray wave front after it undergoes refractive bending through the sample. The color scale unit is radians.

The interferometer consists of three phase gratings arranged along the x-ray beam with approximately equal spacing (Fig. 1a). Their period ratios are approximately 1:1:1 or 2:1:2. The divergent beam from an area source can be viewed as a continuous array of narrow fan beams radiating out from the source. A single fan beam forms a Mach-Zehnder type polychromatic interferometer illustrated in Fig. 1a^16^. The interferograms from the array of fan beams overlap. By the appropriate tuning of the system they are brought into phase to contribute to a single interferogram (Fig. 1b). The tuning includes a small mismatch Δ*D* of the spacing *D* between the gratings and a small difference Δ*P* among the periods of the three gratings, for instance by tilting the first and third gratings. A sample placed in the x-ray field causes differential phase shifts and variable loss of mutual coherence between the interfering light paths, resulting in visible distortions of the interferogram (Fig. 1b). Theoretical modeling of the interferometer and experimental verification are described in the Methods section.

A key requirement of this method is that the period of the central phase grating be less than 2.5 times the lateral coherence of the source at the first grating. Thus, the demand on the coherence of the source is transferred to the phase gratings. For our hard x-ray imaging system the maximum limit on the grating period was 0.4 μm (source size of 60 μm, source-to-first grating distance of 28 cm, central wavelength of 0.41 Å at 40 kVp of tube voltage). Similarly, the demand on detector resolution is also transferred to the phase grating as the interference fringe period scales with 1/(grating period). In our system with 200 nm period gratings the fringe period of the interferogram was 0.50 mm. The effective periods of the first and third gratings were tuned by tilting at an angle *θ*, which gave a cos*θ* factor. For 200 nm gratings a 0.026 radian *θ* reduced the periods by 0.068 nanometers. The grating material and fabrication are detailed in the reference^15^ and summarized in the Methods section. The total distance from the source to the detector was 1.73 m. Other system parameters, the imaging protocol and image reconstruction are also described in the Methods section.

The combination of small grating periods and transparency of the optical elements provide sensitive detection of perturbations of the x-ray wavefront at low radiation dose levels. From a set of raw interferograms, an adaptive algorithm is used to retrieve the phase shift of the interference fringes called a differential phase image^17^ and an image of the de-coherence or scattering of the wavefront^18^,^19^,^20^. The differential phase image is the slope of the phase distribution of the wavefront after it passes through the object, multiplied with an instrumentation factor. It relates to the refraction angle of the x-rays as 2π*(refraction angle)*(sample-to-third-grating distance)/(middle grating period). Figure 1c is an example of a single projection differential phase image of an unstained mouse heart specimen immersed in water, showing the internal anatomy of the heart. When compared to the same image taken with a Talbot-Lau interferometer at twice the radiation dose (entrance surface dose of 1.08 mGray vs. 2.16 mGray) (Fig. 1d), internal anatomical structures become visible owing to an order of magnitude increase in phase contrast. The visible structures include the left and right ventricular walls and the septum, a papillary muscle within the left ventricle, the mitral valve and major blood vessels connected to the heart chambers. The Talbot-Lau grating interferometer is widely used for x-ray tube sources^7^,^10^. It employs absorption gratings of micron-sized slits to filter the source and to resolve a dense interferogram. The level of soft tissue contrast we obtained with the Talbot-Lau system in a single projection is consistent with published results, which used 3D tomography at substantially higher doses to visualize soft tissues^21^,^22^,^23^. More information on the experiment that compared the two systems and quantitative results are presented in the Methods section.

**Figure 2:**
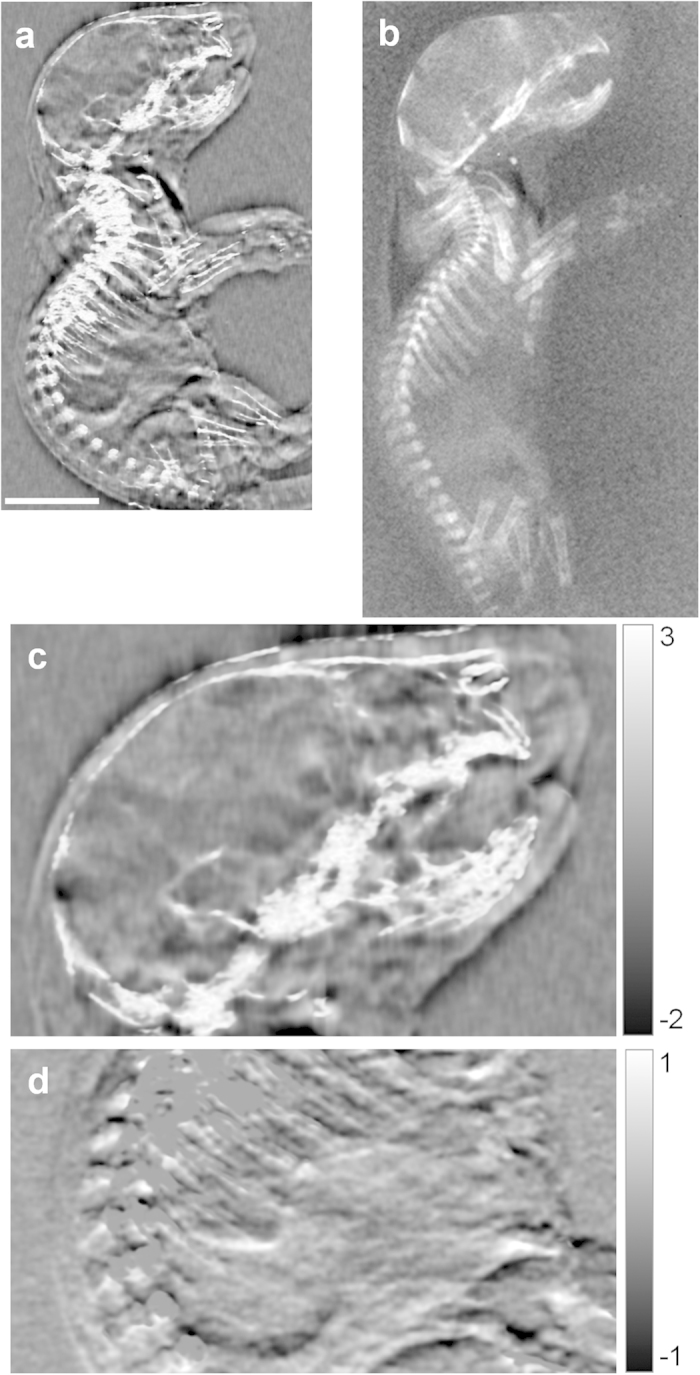
Single-projection phase contrast and attenuation contrast images of a mouse pup specimen suspended in a water-filled chamber. (**a**) Phase contrast image from the PFI at 1.08 mGray entrance surface radiation dose. The phase contrast image is a fused image of the phase shift of the wavefront and the linear intensity attenuation (see Methods section for description). The scale bar is 5.0 mm. (**b**) Attenuation contrast image from a digital flat panel detector at 3.08 mGray dose. (**c**) Magnified view of the head in the fused phase contrast image. Grayscale unit is radians. (**d**) Differential phase data of the abdomen area. Grayscale unit is radians. The highly scattering bones are removed by thresholding the scatter signal.

In another example, an unstained mouse pup specimen was imaged in sagittal projection view. A phase contrast image combining the integrated phase shift and the linear intensity attenuation is shown in Fig. 2a^24^,^25^, with details of the combination described in the Methods section. The phase contrast scan accumulated approximately 1/3 the radiation dose compared with the conventional radiography image (ESD 1.08 mGray vs. 3.08 mGray) (Fig. 2b). In the magnified view of the head region (Fig. 2c), phase contrast showed soft-tissue structures in the brain, the eyes, the nasal cavity and the mouth. The magnified view of the abdomen (Fig. 2d) also revealed detailed structures that are absent in the attenuation contrast image.

**Figure 3:**
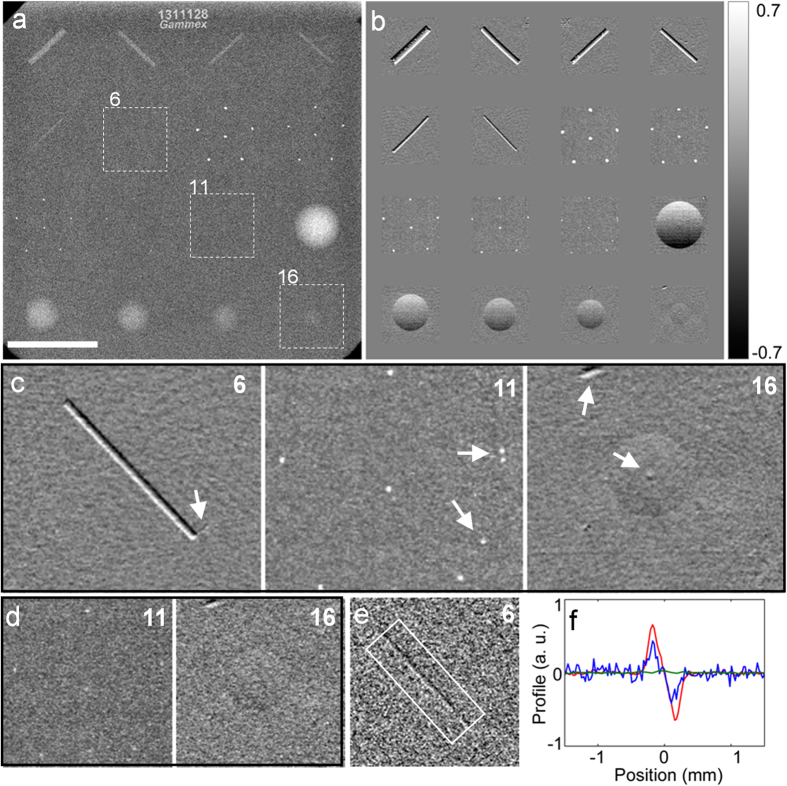
Comparison of images and radiation doses between the interferometer and a digital mammography scanner. A standard mammographic accreditation phantom was studied. (**a**) The digital mammography scanner marginally detected the smallest filament (box 6) and mass (box 16) in the phantom but not the smallest calcification specks (box 11). Scale bar is 2.0 cm. (**b**) A montage of images from the PFI at 78% dose level of the mammography scanner. All features are visible. Grayscale unit is radians. (**c**) Magnified phase contrast views of the smallest features show additional details and defects indicated by arrows. (**d**) At 26% dose level the smallest calcification and mass features are visible with the interferometer. (**e**) The smallest filament seen at 8.1% the dose level. (**f**) Signal profiles across the smallest filament averaged over its length. The red and blue traces are from the interferometer at 78% and 8.1% of the clinical dose level, respectively. The green trace is from the image of the mammography scanner.

To evaluate the potential benefit for human imaging, we imaged a standard phantom used for quality assurance of mammography scanners (Fig. 3). The phantom has three groups of embedded structures that simulate fibrous tissue, micro calcification and tumor masses in the breast, down to 0.16 mm size. We used a high-performance digital mammography scanner as a reference^26^. In a standard clinical protocol on the digital mammography scanner, the smallest fiber (0.4 mm diameter, box 6 in Fig. 3a) and mass (circular lens-shaped feature of 0.25 mm thickness, box 16 in Fig. 3a) were at the detection threshold, while the smallest calcification specks (0.16 mm, box 11 in Fig. 3a) could not be detected. In comparison, the PFI resolved all of the smallest features with additional details and manufacturing defects in the phantom at 78% of the radiation dose (Fig. 3b and magnified in Fig. 3c). At 26% dose level it detected the smallest calcification and mass features (Fig. 3d). The calcification specks were visible in the de-coherence image, indicating that they disrupt the lateral coherence of the wavefront. The smallest fiber feature is still visible at 8.1% the dose level (white box in Fig. 3e). The measured noise floor of the differential phase signal inside the phantom corresponded to a 5.7 nano-radian bending of the x-ray wavefront. Figure 3f shows the differential phase profiles across the smallest fiber at 78% (red) and 8.1% (blue) dose levels, and the conventional attenuation profile (green) at full dose.

## Discussion

Today despite significant progress in developing monochromatic or spatially coherent compact sources^27^,^28^,^29^, the Roentgen vacuum tube is the predominant source in compact imaging systems. In pursuit of better soft tissue detection with x-ray phase contrast techniques we developed an interferometry method using x-ray tube sources and phase gratings only, which also addresses the conflicting demands of detector efficiency versus resolution. The results from the first imaging tests suggest that the interferometer has the potential to detect soft-tissue pathologies at much lower doses than current clinical exams.

Nanometric phase gratings are challenging for current fabrication technology, but the difficulty is tempered by the much shorter path lengths required for phase modulation than intensity modulation. This is due to the fact that in heavy metal elements such as gold, the phase shift of hard x-rays is many times the intensity attenuation. Current gratings have an area of 1.0 mm by 7 cm. Therefore the sample needs to be scanned vertically and the multiple fields of view are stitched together into a full image. As fabrication techniques improve^15^,^30^,^31^, larger area phase masks with better phase profiles can lead to further advances with this method.

## Methods

All animal experiments were performed in accordance with relevant guidelines and regulations, under a protocol approved by the Animal Care and Use Committee of the National Heart, Lung and Blood Institute, National Institutes of Health. A fresh heart specimen was excised from a euthanized adult mouse, fixed in 10% buffered formalin and then immersed in de-ionized water in a 15 mm thick chamber for imaging. A mouse pup was euthanized and prepared in the same procedure for imaging.

### PFI specifications

The interferometer consisted of a tungsten-anode x-ray tube operating at 40 kVp/1.0 mA (Source-Ray Inc., NY, US), three phase-mask gratings of 1:1:1 or 2:1:2 pitch ratios with the central grating pitch of 200 nm, an x-ray detector consisting of a GdOS phosphor screen and a digital camera with a wide-aperture lens^32^ (Nikon D800 SLR, Japan). The grating lines were oriented horizontally. To counter focal-spot drift within the x-ray tube during exposure, a 60 μm-wide tungsten aperture was placed on the tube window. The detector matrix was 1237 × 820 with a pixel size of 52 μm. The detector was tilted to increase the resolution in the direction perpendicular to the grating lines. The inter-grating distance was 46 cm. The distance from the source to the first grating was 28 cm and from the third grating to the detector was 53 cm.

The gratings had an area of 1.0 mm by 7 cm, which set the field of view of a single image. The sample was scanned vertically in 0.6 mm steps and the multiple fields of view were stitched together into full images. The gratings were all rotated about the vertical axis by 60° to increase their effective depths to approximately 7 μm for sufficient phase modulation of the x-ray wave.

The tuning of the system involved adjusting the tilt of the first and third gratings by up to 1° to change their effective periods and adjusting the longitudinal position of the third grating in the range of a few millimeters, which was about 1% of the inter-grating spacing. The interference fringe contrast, defined as Δ*I*_*N*_ = (*I*_*max*_ − *I*_*min*_)/*I*_*average*_, was optimized in this process. The measured and theoretically calculated peak fringe contrasts were 0.281 ± 0.003 and 0.290, respectively. Theoretical modeling predicts a fringe contrast of 0.73 with optimized phase profiles of the gratings. This serves as a guide for grating fabrication development.

### Theoretical model of the PFI and experimental verification

Following the physics model described in the main text we developed a theoretical model called the sum-of-coherent-paths model. Referring to Fig. 1a, A closed-form expression of the interference fringe contrast for a system of central grating period *P* is


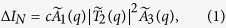


where *c* is a known normalization factor, “∼” represents Fourier transformation, *q* is the Fourier space variable of 2π/*P*, *T*_*j*_ is the complex transmission function of the *j*th grating, and *A*_*j*_ is the shifted auto-product of the *j*th grating (*Ã*_*j*_ being the conjugate of the Wigner distribution)


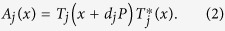


The normalized shifts *d*_*j*_ of the auto-products are determined by the asymmetry in the interferometer, including the deviation Δ*P* of the grating periods and the difference Δ*D* in inter-grating spacings. On the detector, the interference fringe spacing in the direction perpendicular to the grating lines is given by


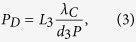


where *L*_3_ is the distance between the third grating and the detector and *λ*_*C*_ is the central wavelength of the x-ray spectrum. In accord with the reciprocal symmetry of electromagnetic wave propagation, the phase of the interferogram at a given location on the detector has a cyclic dependence on the vertical location of the source, with the period of the cycle being


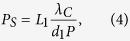


where *L*_1_ is the distance between the source and the first grating. Therefore, for an extended source of size *S*, the grating period should meet the requirement *P*_*S*_ > 2*S*, leading to an upper limit of the grating period


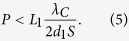


The interference fringe contrast Δ*I*_*N*_ was calculated for the hardware specifications of our system. The calculated Δ*I*_*N*_ versus Δ*D* curves for two tilt angles of the first and third gratings were plotted in supplementary information Fig. 1 together with measured values. They were in good agreement. The calculated interferogram fringe spacing perpendicular to the grating lines was 0.51 mm at the condition of peak fringe contrast, which matched the measured spacing of 0.50 ± 0.03 mm. The maximum allowed grating period from equation (5) was 0.4 μm for our system, which is greater than the actual grating pitch of 0.2 μm.

### Fabrication of hard x-ray phase gratings

The fabrication protocol is described in detail in a previous publication^15^. To briefly summarize, electron beam lithography was used to pattern a master template (Eulitha AG, Switzerland) for nano-imprint lithography on a silicon wafer, from which trenches were etched down to approximately 4 μm via cryogenic reactive ion etching. A conformal layer of platinum was deposited via atomic layer deposition (ALD) followed by conformal electroplating of gold to fill the trenches. Scanning electron micrographs of the cross section of a 200 nm-pitch grating before and after electroplating are shown in supplementary information Fig. 2. In this first iteration the grating area was limited by the length of the electron beam lithography step to 1.0 mm width and 7 cm length. The silicon substrate of each grating was backside-thinned to 170 to 200 μm thickness. Taking into account the 60° rotation of the gratings about the vertical axis, the x-ray path length through the substrate was between 340 and 400 μm.

### X-ray phase-contrast imaging procedure

In the PFI, phase contrast information at each detector pixel was measured by the phase stepping process^9^,^33^. The third grating was stepped in increments of 60 nm. A phase stepping data set included 4 to 12 images of 5 to 20 second exposures, depending on the total radiation dose level. The reported doses are the sum of all exposures. All dose values were measured with an x-ray exposure meter (Rad-Check Plus, Fluke Biomedical, Cleveland, OH). The mouse heart and mouse pup specimens were imaged with a total entrance surface dose (ESD) of 1.08 mGray. The mammographic phantom was the American College of Radiology mammographic accreditation phantom model 156. It was imaged at ESDs of 2.16 to 0.23 mGray, and corresponding average glandular dose (AGD) of 0.96 to 0.10 mGray. The number of images and the exposure time per image were optimized for each dose level. The factors that influenced the choices included mechanical fluctuations in the phase stepping process favoring more images in a phase stepping set to reduce the random chance of under-sampling (degenerate sampling) of the intensity oscillation curve; mechanical drift during long exposures that degraded the interference fringes; the detector dark noise (x-ray off) which was exposure time dependent. For the PFI, 5 images of 5 second exposure each was found to be optimal at the lowest dose level, while 12 images at 20 second exposure to be optimal at the highest dose level. For the Talbot-Lau interferometer, 19 images of 4 second exposure each was found to be optimal at the ESD of 2.16 mGray, while less images of longer exposures did not improve the SNR of the result.

An adaptive image processing method was used to deal with mechanical instability in the interferometer^25^,^34^. It provided images of differential phase, de-coherence and conventional intensity attenuation. The differential phase and the attenuation images were sometimes combined into a phase contrast image. If we define *A*_0_ and Φ as the linear attenuation and the phase shift of the x-ray wavefront after propagation through the sample, *A*_0_ is then the absolute value of the natural logarithm of the transmission, and 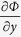 is the differential phase (DP) measurement with the appropriate multiplier, where Y is the direction perpendicular to the grating lines. The first step is to incorporate the derivative of the linear attenuation 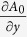 into the DP measurement in a weighted sum of 
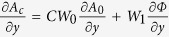
, where *C* is the scaling factor between the real and imaginary parts of the refractive index, and the weights *W*_0_ and *W*_1_ are determined locally according to the amplitude of the interference fringes *A*_1_ and the noise level *N*_1_ in the fringe amplitudes. Once the combined differential image 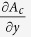
is determined, it is integrated in the Y direction to retrieve a phase shift image which represents the phase distribution of the wavefront in units of radians. Since the initial differential phase measurement from the interferometer is a derivative measurement and lacks low spatial frequency information, the integration process generates smooth varying baseline noise in the Y direction. To counter this problem a high-pass filter in the Y direction is applied.

### Comparison of the PFI with the existing Talbot-Lau interferometer

A Talbot-Lau interferometer was built with the same x-ray source as the PFI. Its source filter and detector analyzer filters were gold absorption gratings of 4.8 μm pitch and 60 μm nominal depths (Microworks GmbH, Germany). The middle phase grating was a silicon π/2 phase-shift grating of 2.4 μm pitch. All gratings were rotated by 30° about the vertical axis for an optimal fringe contrast of 0.40 at 40 kVp. The inter-grating spacing was 39 cm (the third-order Talbot distance for 28 keV). The PaxScan digital flat panel detector was used for image capture. The total length of the system was 1.02 m. A phase stepping set included 19 images of 4-second-exposure each. For quantitative comparison, we measured the differential phase signal in the mammographic phantom with both systems at the same ESD of 2.16 mGray. Images of the largest fiber feature and signal profiles are shown in supplementary information Fig. 3. The level of phase contrast increased by a factor of 15 from the Talbot-Lau system to the PFI. A more detailed description of our Talbot-Lau interferometer and comparison with attenuation radiography is presented in the reference^35^.

### Attenuation contrast radiography

Attenuation contrast radiography of the mammographic phantom was performed with a digital mammography scanner (GE Senographe Essential model). The scanner operated in a standard clinical protocol, at 29 kVp tube voltage and 56 mA*sec in a Rh target/Rh filter configuration, at ESD/AGD of 4.98/1.23 mGray. Its digital flat panel detector has a pixel size of 100 μm. Attenuation contrast radiography of the mouse samples were carried out with a tungsten anode x-ray tube operating at 40 kVp/1.0 mA and a digital flat panel detector (Varian PaxScan 3024M) of 83 μm pixel size. All specimen chambers were placed directly on the surface of the detector to minimize scattering-induced blurring. The ESD was 3.08 mGray.

## Additional Information

**How to cite this article**: Miao, H. *et al.* Enhancing Tabletop X-Ray Phase Contrast Imaging with Nano-Fabrication. *Sci. Rep.* **5**, 13581; doi: 10.1038/srep13581 (2015).

## Supplementary Material

Supplementary Information
