# Supplementary material for: Enhancing Tabletop X-Ray Phase Contrast Imaging with Nano-Fabrication
Source: Sci Rep. 2015 Aug 28;5:13581. doi: 10.1038/srep13581 (PMC4551996; doi:10.1038/srep13581)
Supplement: Supplementary Information [file srep13581-s1.pdf]

**Enhancing Tabletop X-Ray Phase Contrast Imaging with Nano-Fabricatiton**

Houxun Miao[a,1], Andrew A. Gomella[a,1], Katherine J. Harmon[a], Eric E. Bennett[a], Nicholas Chedid[a], Sami Znati[a], Alireza Panna[a], Barbara A. Foster[b], Priya Bhandarkar[b], Han Wen[a, 2]

[a] Imaging Physics Laboratory, Biochemistry and Biophysics Center, National Heart, Lung and Blood Institute, National Institutes of Health, Bethesda, MD 20892

[b] Breast Imaging Center, Walter Reed National Military Medical Center, Bethesda, MD 20889

[1]These authors contributed equally to this work.

[2]To whom correspondence should be addressed. E-mail: wenh@nhlbi.nih.gov

Supplementary Fig. 1

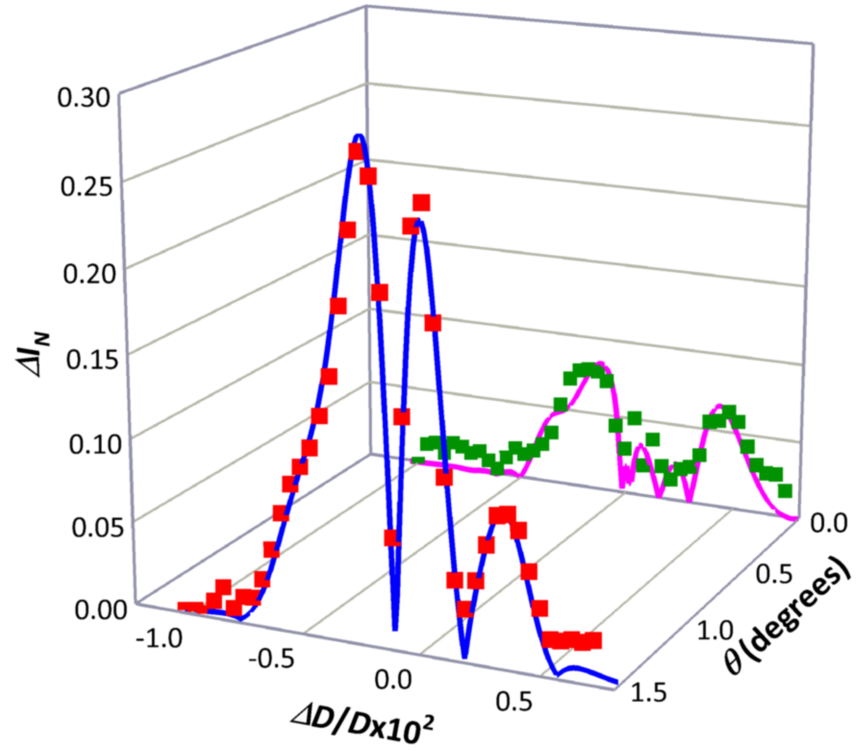


**Supplementary Fig. 1** Theoretical curves of interference contrast and experimental measurements. The theoretical curves (blue and magenta lines) are calculated using Eq. (1) of the Methods section for two tilt angles $\theta$ of the first and third gratings and over a range $\Delta D/D$, which is the percentage difference in inter-grating spacings. The measurements (red and green squares) have a standard deviation of $3\times10^{-3}$.

Supplementary Fig. 2

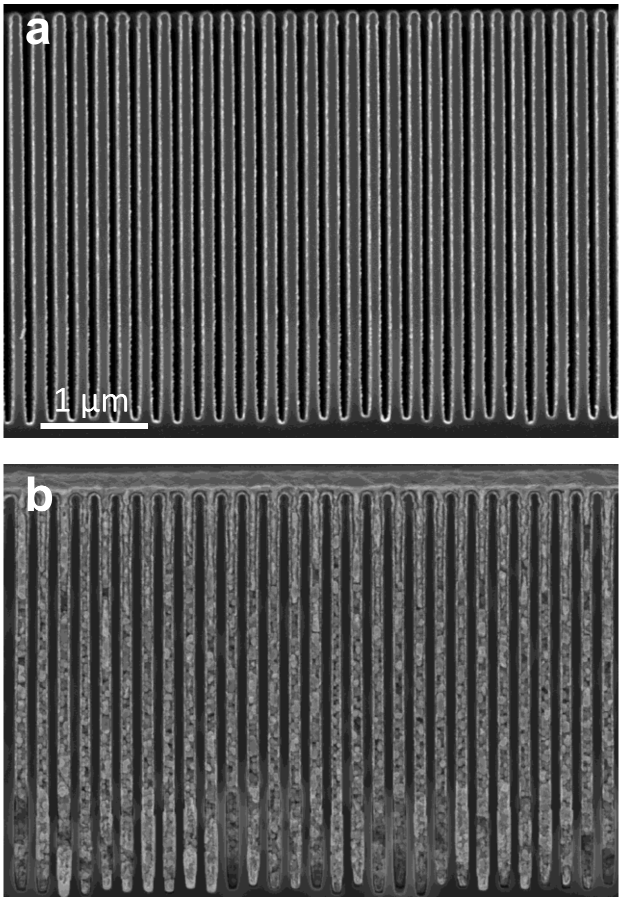


**Supplementary Fig. 2** Illustration of the fabrication of hard x-ray phase grating of 200 nanometer pitch. (**a**) Scanning electron micrograph of the intermediate fabrication stage where the silicon substrate has been coated with a 20 nm platinum layer. (**b**) The trenches are filled with gold via conformal electroplating.

Supplementary Fig. 3

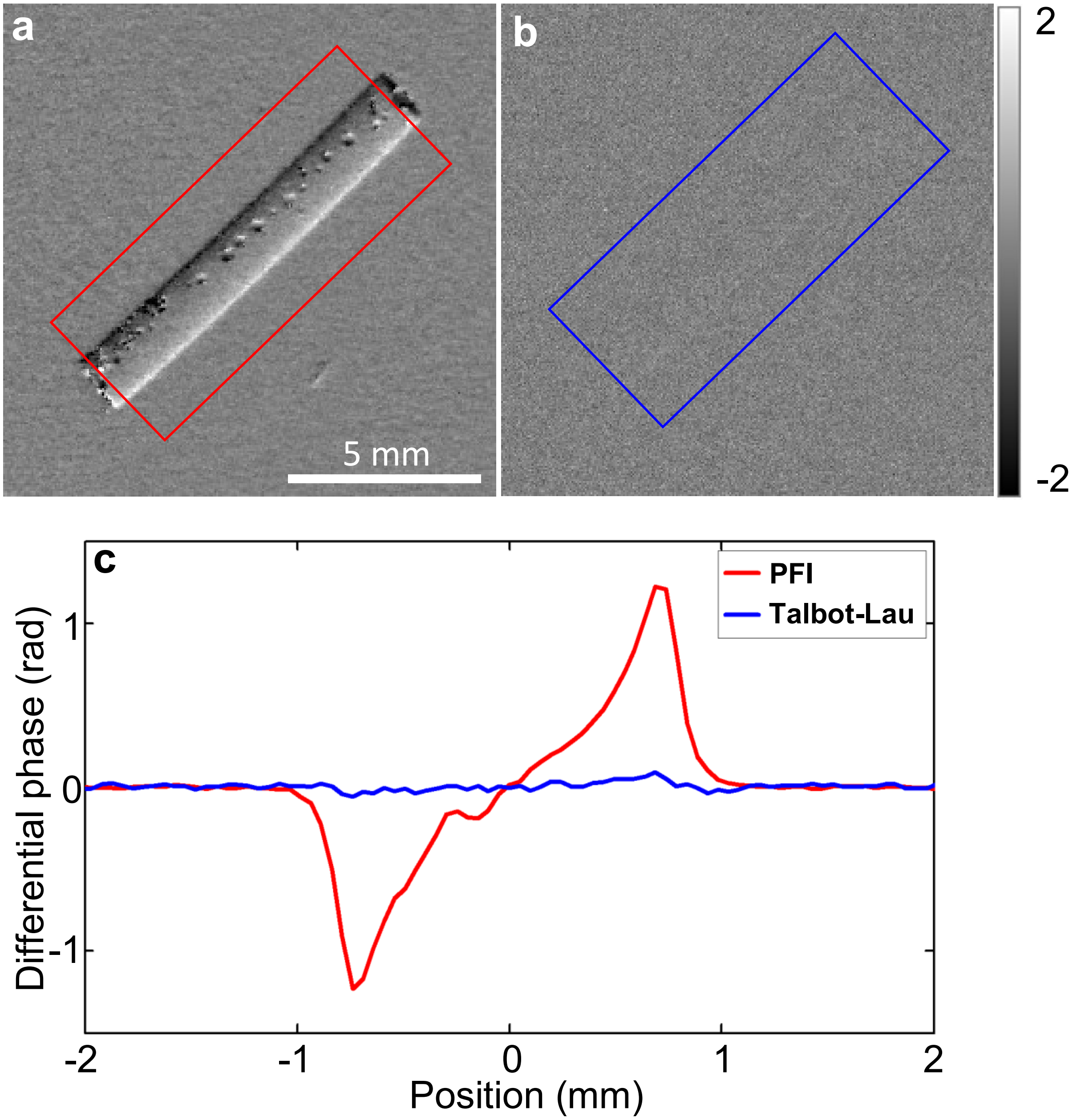


**Supplementary Fig. 3** Comparison of the differential phase signal between a PFI and a Talbot-Lau interferometer of comparable sizes. (**a**) Differential phase image of a nylon fiber in the mammographic phantom from the TRIP interferometer at an entrance surface dose of 2.16 mGray. (**b**) Image from the Talbot-Lau interferometer at the same dose. (**c**) The red trace is the signal profile across the fiber from the TRIP system and the blue trace is from the Talbot-Lau system. The differential phase signal is the measured phase shift of the interference fringes and is proportional to the slope of the transmitted x-ray wave front multiplied by a gain factor of the interferometer.
